# Genetic association of lipids and lipid-lowering drugs with sepsis: a Mendelian randomization and mediation analysis

**DOI:** 10.3389/fcvm.2023.1217922

**Published:** 2023-08-07

**Authors:** Chen Lou, Zhizhen Meng, Yi-Yi Shi, Rui Zheng, Song-Zan Qian, Jingye Pan

**Affiliations:** ^1^ School of The First Clinical Medical Sciences, Wenzhou Medical University, Wenzhou, China; ^2^ Department of Emergency, The Fourth Affiliated Hospital, Zhejiang University School of Medicine, Yiwu, China; ^3^ Department of Anesthesiology, The First Affiliated Hospital of Wenzhou Medical University, Wenzhou, China; ^4^ Department of Critical Care Medicine, Sir Run Run Shaw Hospital, School of Medicine, Zhejiang University, Hangzhou, Zhejiang, China; ^5^ Department of Intensive Care Unit, The First Affiliated Hospital of Wenzhou Medical University, Wenzhou, China

**Keywords:** lipid-lowering drugs, genetic association, sepsis, Mendelian randomization (MR) analysis, lipid

## Abstract

**Background:**

The impact of lipid-lowering medications on sepsis is still not well defined. A Mendelian randomization (MR) study was carried out to probe the causal connections between genetically determined lipids, lipid-reducing drugs, and the risk of sepsis.

**Materials and methods:**

Data on total serum cholesterol (TC), high-density lipoprotein cholesterol (HDL-C), low-density lipoprotein cholesterol (LDL-C), apolipoprotein A-I (ApoA-I), apolipoprotein B (ApoB), and triglycerides (TG) were retrieved from the MR-Base platform and the Global Lipids Genetics Consortium in 2021 (GLGC2021). Our study categorized sepsis into two groups: total sepsis and 28-day mortality of sepsis patients (sepsis28). The inverse-variance weighted (IVW) method was the primary method used in MR analysis. Cochran's Q test and the MR-Egger intercept method were used to assess the heterogeneity and pleiotropy.

**Results:**

In the MR analysis, we found that ApoA-I played a suggestively positive role in protecting against both total sepsis (OR, 0.863 per SD increase in ApoA-I; 95% CI, 0.780–0.955; *P* = 0.004) and sepsis28 (OR, 0.759; 95% CI, 0.598–0.963; *P* = 0.023). HDL-C levels were also found to suggestively reduce the incidence of total sepsis (OR, 0.891 per SD increase in HDL-C; 95% CI, 0.802–0.990; *P* = 0.031). Reverse-MR showed that sepsis28 led to a decrease in HDL-C level and an increase in TG level. In drug-target MR, we found that HMGCR inhibitors positively protected against total sepsis ($\frac{1}{\mathrm{OR}}$, 0.719 per SD reduction in LDL-C; 95% CI, 0.540–0.958; *P* = 0.024). LDL-C and HDL-C proxied CETP inhibitors were found to have a protective effect on total sepsis, with only LDL-C proxied CETP inhibitors showing a suggestively protective effect on sepsis28. In Mediated-MR, BMI exhibited a negative indirect effect in HMGCR inhibitors curing sepsis. The indirect impact of ApoA-I explained over 50% of the curative effects of CETP inhibitors in sepsis.

**Conclusions:**

Our MR study suggested that ApoA-I and HDL-C protected against sepsis, while HMGCR and CETP inhibitors showed therapeutic potential beyond lipid-lowering effects. ApoA-I explained the effects of CETP inhibitors. Our study illuminates how lipids affect sepsis patients and the effectiveness of new drugs, opening new avenues for sepsis treatment.

## Introduction

Sepsis is a significant cause of global mortality, with life-threatening organ dysfunction arising from a dysregulated host response to infection (1). Prompt treatment and resuscitation are essential for managing sepsis and septic shock, with adults presenting with suspected sepsis or septic shock requiring urgent ICU admission within six hours (2). Conventional treatment of sepsis typically involves the use of antibiotics, antiviral drugs, and vasoactive agents. Current drug research mainly focuses on the regulation of inflammation and immune dysfunction to develop new and effective drug therapies for the pathogenesis of sepsis (3). Despite significant progress in the understanding of the mechanisms underlying sepsis, specific drugs for treating sepsis remain unavailable. Therefore, further research is necessary to identify new treatments and approaches for improving the outcomes of patients with sepsis.

Lipids and apolipoproteins are associated with the pathological development of sepsis. Critically ill patients have been reported to experience a significant drop in lipid and lipoprotein levels, which is associated with poor prognosis (4). Moreover, total serum cholesterol (TC), high-density lipoprotein cholesterol (HDL-C), low-density lipoprotein cholesterol (LDL-C), and apolipoprotein concentrations were inversely correlated with interleukin concentrations (5). Previous research highlighted the protective role of HDL in sepsis, including its ability to promote anti-oxidation, anti-apoptosis, lipopolysaccharide (LPS) neutralization, and endothelial protection, in addition to reversing cholesterol transport (6). Apolipoprotein A-I (ApoA-I) and HDL-C, components of HDL, likely played significant roles in these processes. The growing understanding of the role of lipids in sepsis has prompted investigations into the potential effects of lipid-lowering drugs in sepsis. Although lipid-lowering drugs have established benefits in cardiovascular disease and atherosclerosis by lowering LDL-C and triglycerides (TG) concentrations, these benefits did not appear to be associated with improved sepsis. Among these drugs, statins, which inhibit 3-hydroxy-3 methylglutaryl coenzyme A (HMG-CoA) reductase (HMGCR inhibitors), have been shown to protect the vascular endothelium and reduce inflammatory damage (7). Other lipid-lowering drugs, including Niemann-Pick C1-Like 1 inhibitors (NPC1L1 inhibitors), such as ezetimibe, and proprotein convertase subtilisin/kexin type 9 inhibitors (PSCK9 inhibitors), such as evolocumab and alirocumab, also contribute to lowering LDL-C levels. However, cholesteryl ester transfer protein inhibitors (CETP inhibitors), such as anacetrapib, have a more significant impact on increasing HDL-C than on reducing LDL-C and may have therapeutic effects on sepsis (8). Despite the potential benefits of lipid-lowering drugs, there is inadequate evidence of their impact on sepsis.

In previous randomized controlled trials and observational studies on the relationship between lipids and sepsis, it was easy for confounding factors and reverse causality to affect the results of experiments (9). Additionally, conducting these studies involves significant financial and time resources, making it challenging to perform rapid and efficient research on the effects of undiscovered exposures, such as newly developed drugs, on various diseases. Therefore, we employed Mendelian randomization (MR), which uses genetic variants to explore causal associations between risk factors (exposure) and disease (outcome) (10). MR is based on the free combination and distribution of genes during parental meiosis, leading to the random acquisition of genes that are either related or unrelated to genetic variations of the risk factors (10). This phenomenon is similar to the imposition of different experimental conditions for experimental and control groups in a randomized controlled trial. However, external factors do not disturb random allocation under natural conditions. The differences in traits displayed by offspring depend only on genetic variations related to risk factors. This study aimed to clarify the causal connection between lipids, apolipoproteins, lipid-lowering medications, and sepsis by using MR.

## Methods

### Study design

**Figure 1:**
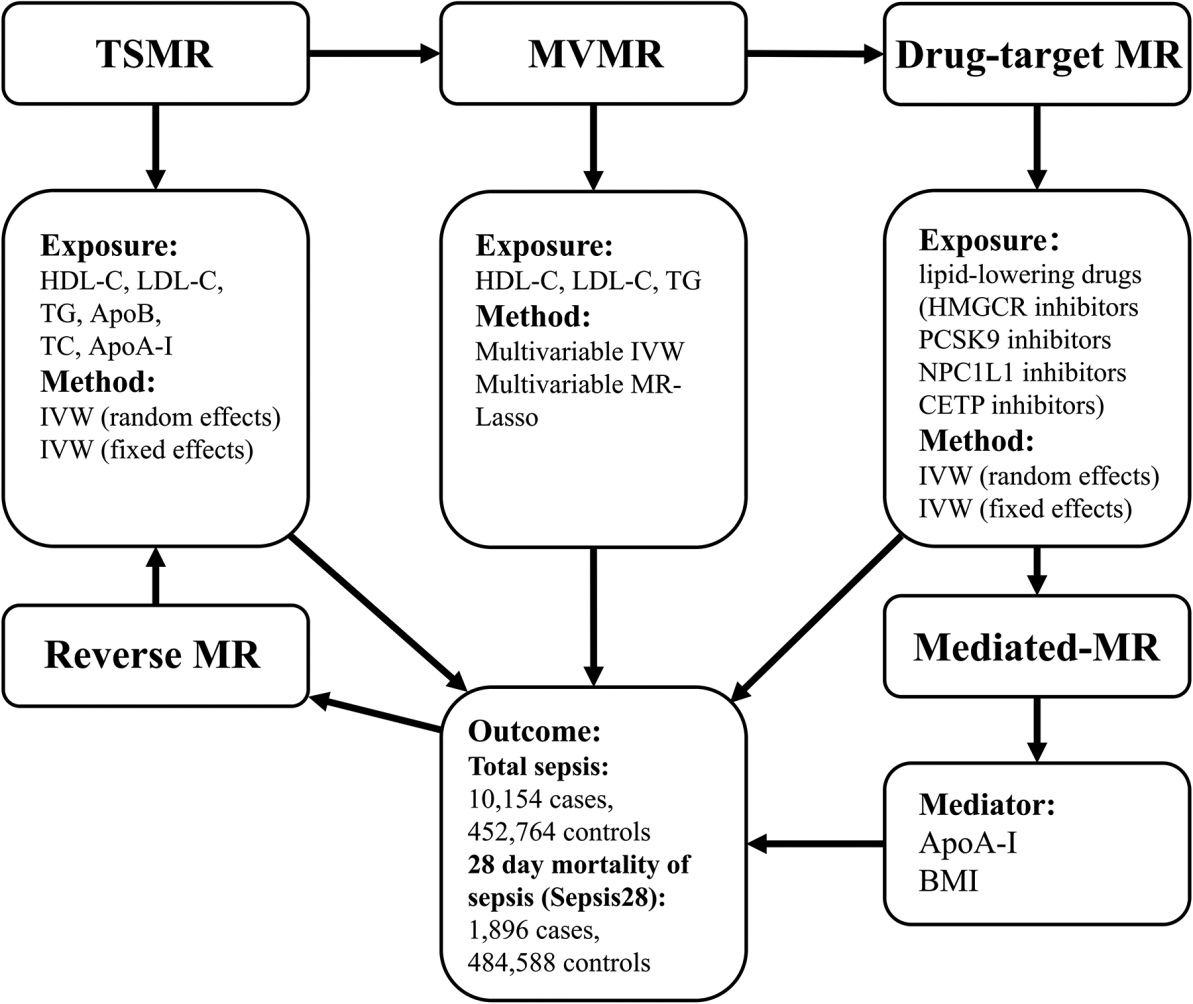
Study design. We carried out TSMR, MVMR, drug-target MR, and reverse MR to clarify the causal relationships between lipids, apolipoproteins, lipid-lowering medications, and sepsis. Afterward, we explored potential intermediate factors through mediated MR. TSMR, two-sample Mendelian randomization; MVMR, multivariable Mendelian randomization; Drug-target MR, drug-target Mendelian randomization; IVW, inverse-variance weighted.

Our investigation adhered to the STROBE-MR guidelines (11), and the S1 STROBE Checklist can be found in the Supporting information. The study flowchart (Figure 1) shows the procedures and substances used. Our first step was to obtain genetic variants (genetic instrument variables (IVs) or single nucleotide polymorphisms (SNPs)) in MR studies to represent lipids and lipid-lowering drugs. During the initial analysis, we used two-sample Mendelian randomization (TSMR) to examine the causality between lipids (HDL-C, LDL-C, TG, TC), apolipoproteins (ApoB, ApoA-I), and sepsis. Next, we employed multivariable Mendelian randomization (MVMR) (12) to analyze the impact of HDL-C, LDL-C, and TG levels on the incidence of sepsis while controlling for the potential confounding effects of the other two variables. Moreover, we employed drug-target Mendelian randomization (drug-target MR) to assess the efficacy of lipid-lowering agents, including HMGCR, NPC1L1, PCSK9, and CETP inhibitors, as treatment options for patients with sepsis. The drug-target MR used SNPs restricted to the locus of a gene coding for the drug target, serving as an indicator of the specific drug (13). In reverse Mendelian randomization (reverse-MR), sepsis acted as the exposure. We examined sepsis's effects on LDL-C, HDL-C, and TG concentrations. Furthermore, we incorporated the mediated Mendelian randomization (mediated-MR) approach to evaluate the potential role of mediators [body mass index (BMI) and ApoA-I] in mediating the relationship between lipid-lowering drugs and sepsis. Our study categorized the outcomes into two groups to better understand sepsis (14). The first category was total sepsis, which included all patients diagnosed with sepsis. The second category, 28-day mortality of sepsis patients (Sepsis28), contained only sepsis cases that resulted in mortality within 28 days. All the experimental groups and data sources are presented in Supplementary Table S1. Supplementary Table S24 explains the abbreviations used.

The MR study required adherence to the three assumptions (10) across the above phases. The first assumption stated that the selected IVs must demonstrate a strong correlation with exposure. The second assumption was that the chosen IVs were not related to any potential confounders between lipid exposure and sepsis outcomes. Finally, the third assumption asserted that IVs must only impact sepsis by risk factors and not through any other pathway (e.g., BMI, age, chronic diseases) (15). This study was conducted in a homogeneous European population, ensuring that the results reflected the genetic characteristics of the population. Using a homogeneous population, we were able to mitigate the impact of extraneous variables and obtain reliable and precise results. Moreover, the possibility of overlap can be reduced by utilizing diverse data sources for exposure and outcome variables, thereby decreasing the potential bias in our MR analysis.

This study employed publicly available data from Genome-Wide Association Studies (GWAS) and existing literature. The underlying primary research has undergone thorough ethical scrutiny and received approval.

### Data source

In this study, different exposures and instrumental variables were chosen for Mendelian randomization. GWAS data for traits, including ApoA-I, and ApoB, were obtained from the metabolite level Quantitative Trait Loci (QTLs) in the MR-Base platform (http://app.mrbase.org/) (16). The data used in this study was initially uploaded by Kettunen et al. To ensure the validity of the results, the authors employed a clustering technique on summary statistics to retain only independent and strongly associated SNPs (17). These data were used to perform TSMR. We obtained another set of data on the lipid levels of TC, HDL-C, LDL-C, and TG from the Global Lipids Genetics Consortium in 2021 (GLGC2021) (https://csg.sph.umich.edu/willer/public/glgc-lipids2021/). GLGC2021 aggregated data from 1,654,960 individuals from 201 primary studies spanning five distinct genetic ancestry groups. Lipid measurements were reported in mg/dl. To eliminate potential biases introduced by sample overlap, meta-analysis summary statistics (*n* = 930,672) were screened to exclude individuals from the UK Biobank study (*n* = 389,344) (18) while ensuring a homogeneous population of European descent. These data were used to perform TSMR and MVMR.

Moreover, lipid data were used to screen the gene targets of drugs with demonstrated effects on lipid levels in preparation for subsequent drug-target MR analysis. As reported in the literature, CETP inhibitors have been shown to significantly increase HDL-C levels and lower LDL-C levels in the human body. Thus, SNPs strongly associated with HDL-C or LDL-C can serve as proxies for CETP inhibitors, which has been reported in many studies (19–21). We used GWAS of HDL-C and LDL-C from GLGC2021 as proxies for CETP inhibitors. Additionally, LDL-C lowering medications, including HMGCR, NPC1L1, and PCSK9 inhibitors, were represented by SNPs associated with LDL-C from GLGC2021 and GLGC in 2013 (GLGC2013) (https://csg.sph.umich.edu/willer/public/lipids2013/) (22, 23) as a verification.

The re-analysis study from which the total sepsis data were derived (24) was a comprehensive investigation conducted using a large sample size comprising 10,154 sepsis cases and 452,764 controls. The sepsis28 data were sourced from a re-analysis study of the IEU GWAS database project (https://gwas.mrcieu.ac.uk/) (16), which included 1,896 cases and 484,588 controls. The unit of measurement employed in these studies was the logOR. These GWAS studies utilized data from the UK Biobank, and the populations analyzed were European and included both male and female individuals. The GWAS of BMI was obtained from a meta-analysis of the Genetic Epidemiology Research on Adult Health and Aging (GERA) cohort and the Genetic Investigation of Anthropomorphic Traits (GIANT) consortium. In total, 331,418 individuals were included in the analysis (25).

### Genetic instrumental variable selection

The SNPs in TSMR were subjected to stringent quality control measures to ensure their genome-wide significance (*P *< 5 × 10^−8^) with exposure and independence from one another (*r*^2 ^< 0.001, *kb* = 10,000) after clumping to minimize linkage disequilibrium (LD). However, to ensure an adequate number of IVs for sepsis, we had to establish another threshold of genome-wide significance in the selected SNPs with a significance level of *P *< 5 × 10^−6^. For MVMR, SNPs that demonstrated a strong association (*P *< 5 × 10^−8^) with one of the lipid traits (HDL-C, LDL-C, or TG) were reserved to form a union of SNPs for the three exposures. SNPs exhibiting a high LD (*r*^2^ > 0.001) were excluded. To select SNPs as proxies for lipid-lowering drugs in the drug-target-MR, we first screened for SNPs that were significantly related (*P *< 5 × 10^−8^) to either HDL-C or LDL-C levels. Among these SNPs, those located close (±100 kb) to the target gene (HMGCR, position:74632154-4657929; PSCK9, position:55505221-55530525; CETP, position:56995762-57017757; NPC1L1, position:44552134-44580914) of each drug were reserved. This screening process has been described in several previous studies (26). Additionally, SNPs with a high LD were removed [*r*^2 ^> 0.1 or *r*^2 ^> 0.3 (23)]. Following the above selection steps, SNPs exhibiting palindromic characteristics that might introduce strand ambiguity were removed from the dataset. We then calculated the size of the F-statistic (Beta^2^/SE^2^) (26) for each SNP and retained only SNPs with an F-statistic greater than 10 to ensure the instrumental strength of the SNPs. For each MVMR exposure, the conditional F-statistic (27) was calculated to exclude weak instrumental variables. Furthermore, SNPs related to potential confounders (*P *< 5 × 10^−8^) were removed using the PhenoScanner R package (28).

### MR analysis

After carefully selecting the instrumental variables (IVs) for exposure and outcome, the TwoSampleMR R package (version 0.5.6) was used for TSMR, drug-target MR analysis, and reverse-MR, with no difference in the analytical methods applied to the two. The inverse-variance weighted multiplicative (IVW) method, comprising inverse-variance weighted multiplicative random effects (IVW-RE), and inverse-variance weighted multiplicative fixed-effects (IVW-FE), was used as the primary analytical strategy (29) to assess the association between exposure and the outcome of sepsis. A causal relationship between exposure and outcome was assumed if the results showed a *P*-value of less than 0.05. However, it was essential to note that the interpretation of the results may differ in drug-target MR analysis. In TSMR, MVMR, and reverse-MR, an OR > 1 indicated that exposure was a risk factor for the outcome. However, in drug-target MR, when using LDL-C as a proxy to conduct MR analysis on drug targets, an OR > 1 indicated that an increase in LDL-C level was a risk factor for the outcome. However, the exposure factor studied was a lipid-lowering drug that acted on a drug target to inhibit the increase in LDL-C. Therefore, the results should be interpreted in reverse, meaning that the actual effect of the lipid-lowering drug on sepsis outcomes was the reciprocal of the original OR value ($\frac{1}{\mathrm{OR}}$). $\frac{1}{\mathrm{OR}}<1$ implied that the use of lipid-lowering drugs might lower the risk of the outcome. It was worth noting that if the medicine being studied was a CETP inhibitor, which increased HDL-C and decreased LDL-C, then the conversion process of the OR values described above would be unnecessary when using HDL-C as a proxy for the CETP gene.

For Mediated-MR, we first conduct a two-by-two TSMR analysis of the exposure, outcome, and mediator. We employed the product of coefficients method using the results from two separate MR analyses between the exposure and mediator (Beta_1_, SE_1_) and the mediator and outcome (Beta_2_, SE_2_). Using this method, we evaluated the indirect effect (Beta = Beta_1_ * Beta_2_, SE = sqrt(beta1^2 * se2^2 + beta2^2*se1^2 + se1^2*se2^2) and assessed its statistical significance (30). The direct impact was calculated by subtracting the indirect effect (established using the product of coefficient method) from the total effect (obtained from the TSMR analysis).

Cochran's Q test was used to assess the SNP heterogeneity (31). This test encompasses MR-Egger and inverse variance-weighted methods. If the test showed a *P*-value less than 0.05, indicating significant heterogeneity among SNPs, the IVW-RE method was employed to correct for potential biases. Otherwise, the IVW-FE method was used for the MR analysis. The MR-PRESSO R package (32) was used to identify and remove the heterogeneous SNPs from the dataset. The MR-Egger intercept method (32) was employed to evaluate pleiotropy, and a *P*-value of less than 0.05 indicated significant pleiotropy. The Mendelian randomization R package (version 0.5.1) and MVMR R package (27) were used for MVMR. The primary analytical methods employed were the multivariable IVW and MR-Lasso methods. Conventional Q-statistics estimated the horizontal pleiotropy and heterogeneity of MVMR. In order to compensate for multiple tests, we employed the Bonferroni adjustment to modify the significance level thresholds. As such, a strong evidence was indicated for *p* < 0.05/x/y, where × represents exposures and y signifies outcomes. Furthermore, a suggestive evidence was proposed for 0.05/x/y ≤ *p* < 0.05. All data analyses were conducted using R software (version 4.2.2).

## Results

### Instrumental variable selection

For the SNPs utilized in the TSMR and MVMR analyses, we ensured that all selected SNPs were significantly associated with the exposures of interest, independent of each other, and not weak instruments (*F* > 10). For exposures whose outcome was sepsis, we removed SNPs strongly associated with potential confounding factors, including body mass index, body composition traits, cancer, diabetes, drug allergic reactions, intestinal obstruction, chronic liver or kidney disease, and inflammatory cytokines (Supplementary Table S22). The results of the MR-PRESSO test are presented in Supplementary Table S21. Finally, we selected SNPs from the GLGC2021 and MR-Base databases. Specifically, we selected 326, 201, 249, and 215 SNPs to represent TC, LDL-C, HDL-C, and TG, respectively (Supplementary Table S2), from the GLGC2021 data. We also removed the SNPs that were strongly interrelated and confounded among LDL-C, HDL-C, and TG. Additionally, we selected 7, and 11 SNPs from the MR-Base database to represent, ApoA-I, and ApoB, respectively (Supplementary Table S2). In MVMR, we obtained 322 SNPs representing exposures, namely LDL-C, HDL-C, and TG (Supplementary Tables S5, S6). The lowest conditional F-statistic for a single exposure factor was 34.452. Furthermore, to investigate the causal effect of sepsis on lipid concentrations in reverse-MR, we selected 23 and 14 SNPs to represent total sepsis and sepsis28, respectively (Supplementary Table S12).

### Causality between lipids and sepsis

**Figure 2:**
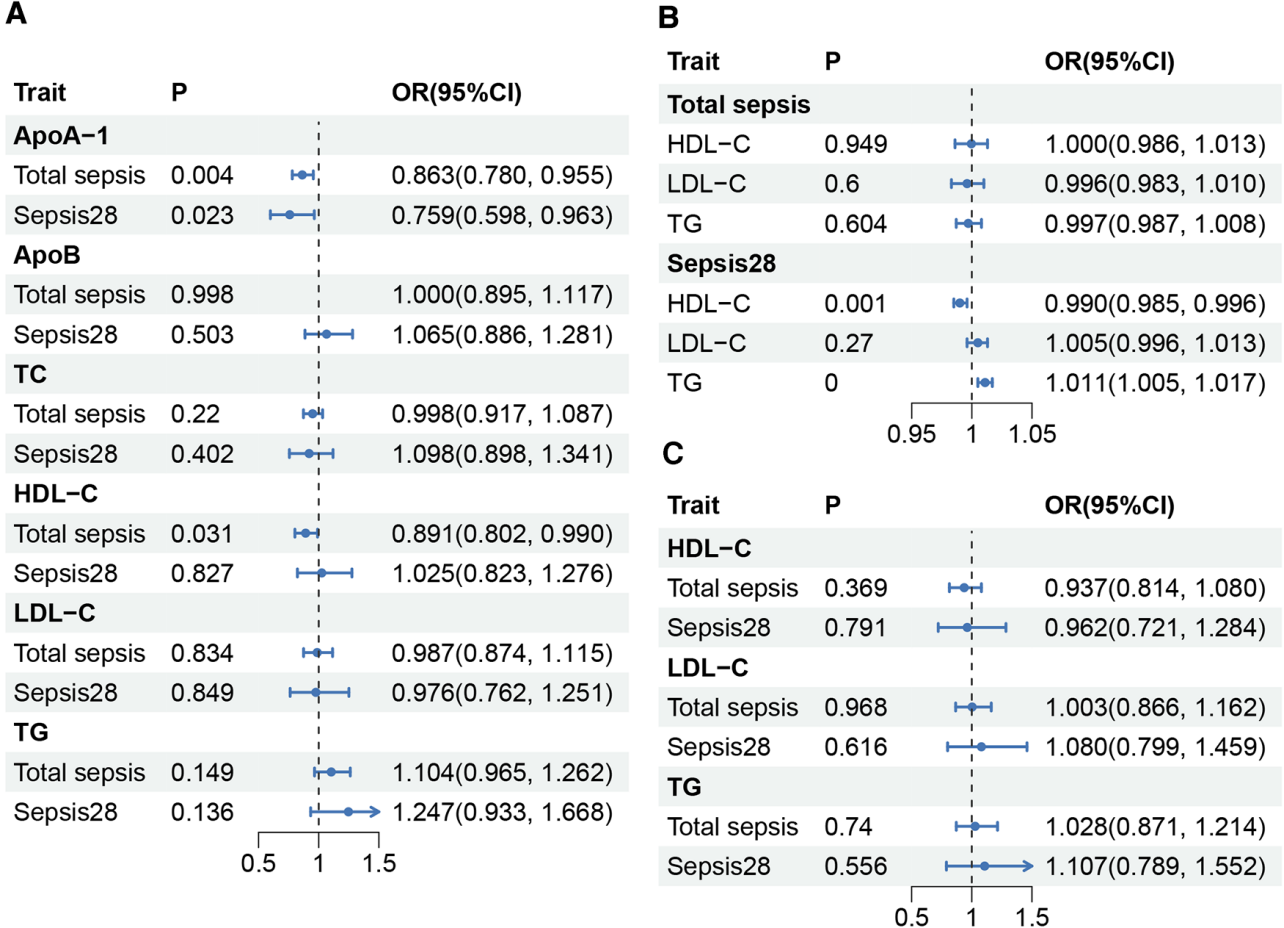
Causality between lipids and sepsis. (**A**) Results of TSMR between sepsis and lipids. ApoA-I, and ApoB were obtained from the MR-Base platform, while TC, HDL-C, LDL-C, and TG were obtained from GLGC2021. (**B**) Results of reverse-MR between sepsis and HDL-C, LDL-C, and TG. (**C**) Results of MVMR between sepsis and HDL-C, LDL-C, and TG. HDL-C, total cholesterol in HDL; LDL-C, total cholesterol in LDL; TG, serum total triglycerides; TC, serum total cholesterol; P, *P*-value; OR, odds ratio; CI, Confidence interval.

The main MR results are shown in Figure 2. The effects of apolipoproteins were investigated using TSMR analysis, which showed that ApoA-I plays a suggestively positive role in the protective effects against total sepsis (OR, 0.863 per SD increase in ApoA-I; 95% CI, 0.780–0.955; *P* = 0.004) and sepsis28 (OR, 0.759; 95% CI, 0.598–0.963; *P* = 0.023), whereas no correlation was observed between ApoB and sepsis. We found that the elevation of HDL-C levels was suggestively beneficial for reducing the risk of total sepsis (OR, 0.891 per SD increase in HDL-C; 95% CI, 0.802–0.990; *P* = 0.031), but had no protective effect on sepsis28 (OR, 1.025; 95% CI, 0.823–1.276; *P* = 0.827). TC, TG, and LDL-C levels were not found to be associated with sepsis (Supplementary Tables S3, S4). Thus, TSMR supported the active role of ApoA-I in protecting against sepsis and sepsis28 and the role of HDL-C in protecting against sepsis. Subsequently, we investigated the effects of sepsis on lipid levels using reverse-MR. Our results (Supplementary Table S13) showed that total sepsis did not significantly affect lipid content in the body. However, sepsis 28 led to a significant decrease in HDL-C levels (OR, 0.99; 95% CI, 0.985–0.996; *P* = 0.001) and an significant increase in TG levels (OR, 1.011; 95% CI, 1.005–1.017; *P* = 1.84E-04). This result illustrates the possible effect of sepsis28 on the lipid content in the body. To corroborate the protective effect of ApoA-I on sepsis, we replicated the analysis using a larger ApoA-I GWAS summary statistics (Open GWAS ID: ieu-b-107, current sample size of *N* = 393,193). Due to the lack of larger non-UK Biobank data sets, we used the ApoA-I dataset in UK Biobank for replication, despite the sample overlap limitations. The results of this replication MR study were similar with the previous positive ApoA-I results (Supplementary Table S23).

In MVMR, after controlling for the effects of TG and LDL-C, the IVW method indicated that an increase in HDL-C level was not significantly associated with either total sepsis (OR, 0.937; 95% CI, 0.814–1.080; *P* = 0.369) or sepsis28 (OR, 0.962; 95% CI, 0.721–1.284; *P* = 0.791). Furthermore, the TG and LDL-C levels did not affect sepsis improvement. We conducted an additional validation using the MR-Lasso method because of the significant heterogeneity observed in the total sepsis outcome group. The results from the MR-Lasso method were consistent with those results using the IVW method (Supplementary Tables S7, S8). We found no association between sepsis and any other type of lipid after controlling for the effects of the other two lipid factors.

### Causality between lipid-lowering drugs and sepsis

Following previously described criteria, we screened SNPs in genes adjacent to the drug target of interest to serve as proxies for lipid-lowering drugs (21, 24). Using LDL-C data from GLGC2021, we identified 15, 8, 23, and 5 SNPs as proxies for HMGCR, NPC1L1, PSCK9, and CETP inhibitors, respectively. Using HDL-C data, we identified 16 SNPs (with the lowest F-statistic of 43.378) as proxies for CETP inhibition using an alternative approach. Using LDL-C data from GLGC2013, we identified 7, 3, and 11 SNPs as proxies for HMGCR, NPC1L1, and PSCK9, respectively. Detailed information on all the SNPs is presented in Supplementary Table S9. During the screening process for SNPs that might be associated with confounding factors, we found that several SNPs near HMGCR (rs12916, rs3804231, rs10066707, rs72633962, and rs1544755) were associated with BMI, but SNPs were not found to be associated with other confounding factors. Therefore, we excluded SNPs associated with BMI and obtained 3 and 14 SNPs to serve as proxies for HMGCR inhibitors screened in GLGC2013 and GLGC2021, respectively. We referred to these newly screened groups as “HMGCR-correct inhibitors”.

**Figure 3:**
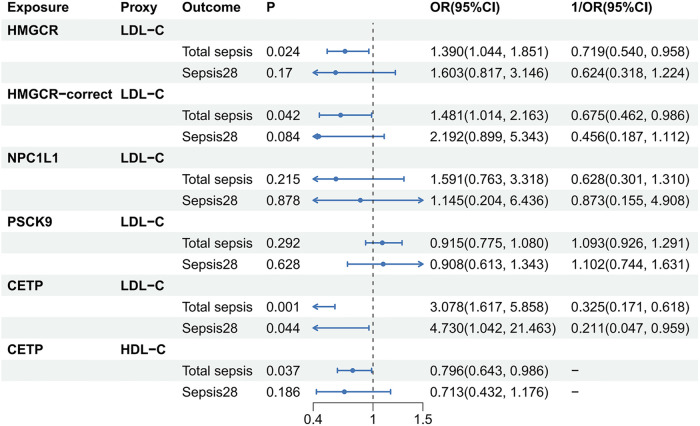
Causality between lipid-lowering drugs and sepsis. The source of lipids used to act as lipid-lowering drugs here was GLGC2021. HDL-C, total cholesterol in HDL; LDL-C, total cholesterol in LDL; P, *P*-value; OR, odds ratio; CI, Confidence interval.

The results are shown in Figure 3. Based on data from GLGC2021, we found that HMGCR inhibitors ($\frac{1}{\mathrm{OR}}$, 0.719 per SD reduction in LDL-C; 95% CI, 0.540–0.958; *P* = 0.024), HMGCR-correct inhibitors ($\frac{1}{\mathrm{OR}}$, 0.675 per SD reduction in LDL-C; 95% CI, 0.462–0.986; *P* = 0.042), LDL-C proxied CETP inhibitors ($\frac{1}{\mathrm{OR}}$, 0.325 per SD reduction in LDL-C; 95% CI, 0.171–0.618; *P* = 0.001), and HDL-C proxied CETP inhibitors (OR, 0.796 per SD increase in HDL-C; 95% CI, 0.643–0.986; *P* = 0.037) had a protective effect on total sepsis. However, only LDL-C proxied CETP inhibitors showed a suggestively protective effect against sepsis28 ($\frac{1}{\mathrm{OR}}$, 0.211 per SD reduction in LDL-C; 95% CI, 0.047–0.959; *P* = 0.044). NPC1L1 and PSCK9 did not cure or worsen sepsis. Similar results were obtained using the GLGC2013 data (Supplementary Tables S10, S11). Overall, we found that HMGCR and CETP inhibitors had positive effects in the treatment of total sepsis. However, the therapeutic effects of HMGCR and CETP inhibitors in sepsis28 were not robust.

### Indirect effects of mediators on the treatment of sepsis with lipid-lowering drugs

We conducted mediated-MR to elucidate the mediating role of BMI in the relationship between HMGCR inhibitors and sepsis. Specifically, we investigated the effects of HMGCR inhibitors on BMI and the impact of BMI on sepsis. We identified 123 SNPs that were representative of BMI (Supplementary Table S14). Our findings suggest that HMGCR inhibitors may increase BMI from a genetic perspective ($\frac{1}{\mathrm{OR}}$, 1.261 per SD reduction in LDL-C; 95% CI, 1.192–1.333; *P* = 5.44E-16), and that an elevated BMI is detrimental to sepsis development (OR, 1.208; 95% CI, 1.061–1.377; *P* = 0.004). Ultimately, we observed a direct effect (beta = 0.373) and an indirect effect in the opposite direction (beta = −0.044, *P* = 0.006) between HMGCR inhibitors and sepsis. Data from GLGC2013 yielded similar results (Supplementary Table S16). Considering the adverse effects of BMI as a mediating factor, we concluded that the therapeutic implications of HMGCR inhibitors might be more significant than initially thought.

**Figure 4:**
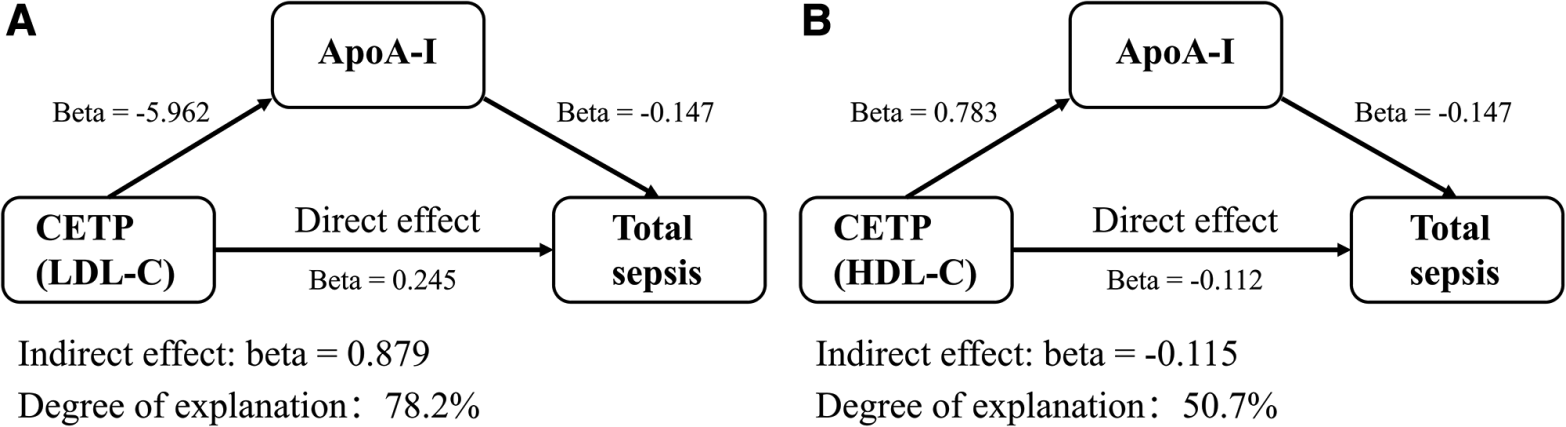
Indirect effects of mediators on the treatment of sepsis with lipid-lowering drugs. The source of lipids used to proxy was GLGC2021. (**A**) ApoA-I explained a part of LDL-C proxied CETP inhibitors’ effects on sepsis. (**B**) ApoA-I explained a part of HDL-C proxied CETP inhibitors’ effects on sepsis. OR, odds ratio; BMI, body mass index.

To better understand the therapeutic effects of CETP inhibitors on sepsis, we analyzed ApoA-I as a mediator. We found that CETP inhibitors proxied by HDL-C significantly increased ApoA-I levels (OR, 2.187 per SD increase in HDL-C; 95% CI, 1.919–2.493; *P* = 1.09E-31). The directions of the indirect effect (Beta = −0.115, *P* = 0.006) and the total effect (Beta = −0.228, *P* = 0.037) were the same. The indirect effect explained approximately 50.7% of the total effect, whereas the remaining effects might occur through other pathways. Indirect effect of ApoA-I was also observed in the group of CETP inhibitors proxied by LDL-C and explained approximately 78.2% of the total effect (Figure 4, Supplementary Tables S17, S18).

## Discussion

**Figure 5:**
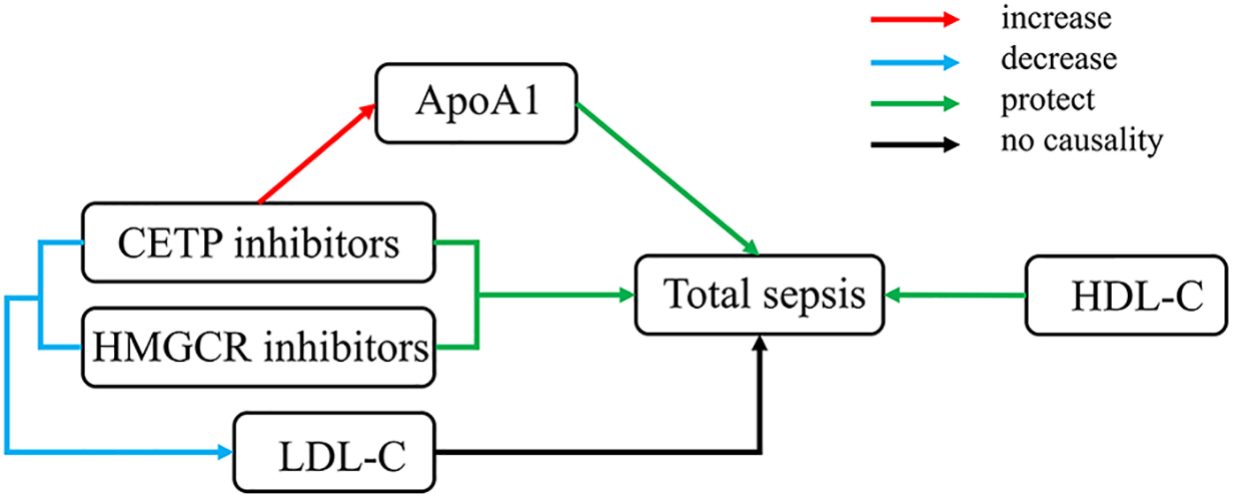
Overall experimental results. HDL-C, total cholesterol in HDL; LDL-C, total cholesterol in LDL.

Our MR study suggested that ApoA-I and HDL-C had protective effects against sepsis, while HMGCR and CETP inhibitors had therapeutic potential beyond their lipid-lowering function. However, the exact role of HDL-C level remains unclear. We also pointed out that the therapeutic effect of HMGCR inhibitors might decrease because of the indirect effect of BMI. Additionally, ApoA-I accounts for over 50% of the effects of CETP inhibitors on sepsis. The experimental results are shown in Figure 5.

ApoA-I and HDL-C are components of HDL, and their functions represent HDL's role in sepsis. Our research reinforces previous studies that demonstrated the beneficial effects of ApoA-I in sepsis. ApoA-I was associated with increased 30-day mortality in sepsis and platelet activation (33). Mice lacking ApoA-I were more susceptible to septic death, with a survival rate of only 47.1%, whereas the survival rate of the control group was 76.7%. In contrast, mice with an increased level of ApoA-I showed resistance to sepsis (34). Our study also revealed a decrease in HDL-C concentration in reverse-MR, consistent with prior research (5, 35), as a consequence of severe sepsis. A low-lipoprotein phenotype, characterized by reduced HDL-C and ApoA-I levels, has been associated with endothelial dysfunction, organ failure, and poor prognosis (4). Synthetic HDL (sHDL) supplementation was reported to improve kidney function, reduce inflammation, and protect against sepsis (36). The protective effects of ApoA-I and HDL-C that our study found support the above view. However, we found that the protective effect of HDL-C was not notably robust in MVMR, possibly because of the method of measurement, which entailed quantifying cholesterol concentration in the non-precipitable lipoprotein fraction of plasma (6). Other cholesterol components might also influence the efficacy of this approach. Also, the MVMR analysis does not necessarily disprove the protective effect of HDL-C, due to its low statistical power. Additionally, the functions of HDL cannot be fully represented solely by HDL-C levels, as different HDL structures, sizes and components can also have an effect (37, 38). One particularly noteworthy point is that lipoproteins are categorized into distinct subgroups based on diameter, with HDLs divided into four sizes: small, medium, large, and extra-large (S-HDL, M-HDL, L-HDL, XL-HDL), and LDLs into S-LDL, M-LDL, and LDL-L through NMR. Certain outcomes were associated with specific lipoprotein subfractions. Hence, the observed HDL-C and sepsis correlation could be influenced as the HDL-C measure encapsulates multiple HDL subfractions. Given that HDL lipoproteins are linked to various anti-inflammatory factors, it is highly probable that the association between HDL-C and sepsis is attributed to HDL functionality rather than HDL-C alone.

Our study did not find associations between LDL-C, TG and sepsis, which have rarely been mentioned in the literature. However, this result also shows that the effect of lipid-lowering drugs on sepsis cannot be achieved by reducing the LDL-C and TG levels. The positive effects of statins were explained as pleiotropic effects independent of lipid-lowering effects, namely, anti-inflammatory and immunomodulatory effects (39). Our finding regarding statin therapy for sepsis was consistent with many clinical trials that have been conducted. Previous studies have reported decreased levels of the inflammatory cytokines TNF-α and IL-6 following oral administration of simvastatin in patients with acute bacterial infection (40). The ASEPSIS Trial found that administering 40 mg/day atorvastatin to patients with sepsis who had not previously taken statins can significantly reduce the conversion rate to severe sepsis. This finding suggested that atorvastatin may have preventative effects against sepsis (41). Another multicenter randomized trial discovered that prior statin use was associated with lower IL-6 levels and that continuing atorvastatin was associated with improved survival (42). However, several SNPs that proxied HMGCR inhibitors were strongly associated with BMI. The use of mediated-MR revealed that BMI mediated the adverse effects of HMGCR inhibitors on sepsis. Previous studies have reported that statins increase the risk of type 2 diabetes and body weight, which were considered risk factors for sepsis (43). Additionally, elevated BMI was linked to increased susceptibility to skin infections, urinary tract infections, and sepsis (44). Therefore, to eliminate confounding effects related to BMI, we conducted an MR study on the HMGCR-correct group and observed the therapeutic effects of HMGCR inhibitors on sepsis. This finding further confirms the positive role of statins in sepsis. However, further research is necessary to determine how to adjust for adverse effects of BMI and other factors during treatment.

For CETP inhibitors, we used LDL-C and HDL-C as proxies and observed their protective effects against sepsis. Previous research has indicated that individuals carrying the A allele of the CETP gene variant (rs1800777-A) experience reduced HDL-C levels during sepsis. This resulted in elevated mortality rates, increased organ failure, and a greater need for organ support compared to those who did not carry the allele (45). Reduction in plasma CETP on day three of sepsis has been linked to mortality (46). Moreover, in mouse models, anacetrapib, a CETP inhibitor, reduced the severity of endotoxemia and improved survival by preserving the HDL-C and ApoA-I. These findings suggested that CETP inhibitors effectively slow the breakdown of HDL particles and prevent the formation of dysfunctional acute-phase HDL during sepsis (47). This function prevents the occurrence of endotoxemia by effectively binding to lipopolysaccharide and triggering a proinflammatory response throughout the body's macrophages, aiding in the elimination of bacterial infections (8). In our research, both the LDL-C and HDL-C proxied CETP inhibitors, which lower LDL-C and elevate HDL-C, can treat sepsis by increasing ApoA-I. The shared emphasis from both proxies underscores the significant mediating role of ApoA-I, each with an explanatory power exceeding 50%. This confirms the viewpoint that it is the components related to HDL, rather than other pathways, that play a protective role in sepsis. One crucial point is that torcetrapib, another CETP inhibitor, was discovered to increase the risk of cardiovascular mortality, cancer, and sepsis. However, these adverse effects were believed to be unique to torcetrapib and were caused by off-target effects such as aldosterone retention and elevated blood pressure (48). Therefore, more extensive randomized controlled trials in humans are required to explore the role of specific lipid-lowering drugs in sepsis.

Our study has several strengths. First, we used Mendelian randomization to overcome potential external confounding factors that often interfere with traditional randomized controlled studies. In this study, we systematically explored the correlation between lipids, apolipoproteins, lipid-lowering drugs, and sepsis, and discovered new therapeutic values for lipid-lowering medications. To minimize bias and clarify the direct or indirect relationship between exposure and outcome, we adopted several MR methods, including TSMR, MVMR, and mediated-MR. Moreover, we sourced data from multiple European population databases, including GLGC and MRbase, to ensure the reproducibility of our results through repeated experiments. For CETP drug targets, we employed different proxy methods to increase the robustness of our findings.

Some limitations of the MR study still need to be acknowledged. Some inherent problems of Mendelian randomization are weak instrumental variables and the difficulty in fully representing traits. In addition, our study population was limited to the European population. Therefore, to confirm our findings, we need to expand the sample size to include individuals of different races. The functions of other subfractions and components of HDL are not fully covered in this paper, and will be further explored in subsequent research. It is important to note that individuals may suffer from sepsis differently, and the two categories of sepsis in this study may require further refinement.

## Conclusion

Our MR study revealed that ApoA-I and HDL-C had protective effects against sepsis. However, the role of HDL-C was not robust. In drug-MR, HMGCR and CETP inhibitors showed therapeutic value for sepsis. These effects might be independent of the lipid-lowering effects of these drugs. We also found an indirect detrimental effect of BMI on treating sepsis by HMGCR inhibitors. ApoA-I was also found to explain half of the effects of CETP inhibitors on sepsis. Our research sheds light on the impact of lipids on patients with sepsis and the efficacy of novel therapeutic drugs, thereby offering new prospects for treating sepsis. Further investigations should focus on the mechanisms of the association among lipids, lipid-lowering drugs, and sepsis.

## Data Availability

The original contributions presented in the study are included in the article/**Supplementary Material**, further inquiries can be directed to the corresponding authors.
