# Supplementary material for: Genetic association of lipids and lipid-lowering drugs with sepsis: a Mendelian randomization and mediation analysis
Source: Front Cardiovasc Med. 2023 Aug 7;10:1217922. doi: 10.3389/fcvm.2023.1217922 (PMC10446761; doi:10.3389/fcvm.2023.1217922)
Supplement: Supplementary file 2 [file Datasheet2.docx]

**Main codes used in this study.** This document includes the code used in TSMR, MVMR, and the code for filtering drug targets. We also included a code for removing confounders. The code for drug-target MR is similar to that for TSMR.

#TSMR, drug-target MR, reverse MR

harmon_ex<-read.csv("harmon.csv")

res_ex<- mr(harmon_ex,method_list=c('mr_ivw_mre','mr_ivw_fe'))

OR <-generate_odds_ratios(res_ex)

OR

#heterogeneity test

het_ex <- mr_heterogeneity(harmon_ex)

het_ex

#pleiotropy test

pleio_ex <- mr_pleiotropy_test(harmon_ex)

pleio_ex

#MR-PRESSO test

library(MRPRESSO)

mrpresso<-mr_presso(BetaOutcome = "beta.outcome",

BetaExposure = "beta.exposure",

SdOutcome ="se.outcome",

SdExposure = "se.exposure",

OUTLIERtest = TRUE,

DISTORTIONtest = TRUE,

data = harmon_ex,

NbDistribution = 1000,

SignifThreshold = 0.05)

mrpresso

#MVMR

library(MendelianRandomization)

library(TwoSampleMR)

library(MVMR)

exp<-read.csv("harmon.csv")

r_input <- mr_mvinput(

bx = as.matrix(exp[,c("Beta_HDL","Beta_LDL","Beta_TG")]),

by= exp$Beta_outcome,

bxse = as.matrix(exp[,c("SE_HDL","SE_LDL","SE_TG")]),

byse = exp$SE_outcome,

snp = exp$SNP,

exposure = c("HDL","LDL","TG"),

outcome = "Sepsis")

#MR-Lasso

lasso<-mr_mvlasso(

r_input,

orientate = 1,

distribution = "normal",

alpha = 0.05,

lambda = numeric(0)

)

lasso

#IVW

mvivw<-mr_mvivw(r_input,model="default",robust = FALSE,correl = FALSE,distribution = "normal",alpha = 0.05)

mvivw

pleres <- pleiotropy_mvmr(r_input = F.data, gencov = 0)

pleres

#calculate the F statistic

F.data <- format_mvmr(BXGs = cbind(exp$Beta_HDL,exp$Beta_LDL,exp$Beta_TG),

BYG =exp$Beta_outcome,

seBXGs = cbind(exp$SE_HDL,exp$SE_LDL,exp$SE_TG),

seBYG = exp$SE_outcome,

RSID = exp$SNP)

strengthres <- strength_mvmr(r_input = F.data, gencov = 0)

strengthres

#select SNPs for LDL-C proxied drug targets

LDL<-fread("without_UKB_LDL_INV_EUR_HRC_1KGP3_others_ALL.meta.singlevar.results.gz")

LDL$P<-2*pnorm(-abs(LDL$EFFECT_SIZE / LDL$SE))

exp_LDL<-subset(LDL,P<5e-08)

head(exp_LDL)

colnames(exp_LDL)[colnames(exp_LDL)=="rsID"] <- "SNP"

colnames(exp_LDL)[colnames(exp_LDL)=="P"] <- "pval.exposure"

HMGCR<-subset(exp_LDL,POS_b37<74757929)

HMGCR<-subset(HMGCR,POS_b37>74532154)

a=clump_data(HMGCR,clump_kb = 10000,

clump_r2 = 0.1)

HMGCR<-merge(a,HMGCR,by="SNP")

write.csv(HMGCR,file="HMGCR.csv")

NPC1L1<-subset(exp_LDL,POS_b37<44680914)

NPC1L1<-subset(NPC1L1,POS_b37>44452134)

a<-clump_data(NPC1L1,clump_kb = 10000,

clump_r2 = 0.1)

NPC1L1<-merge(a,NPC1L1,by="SNP")

write.csv(NPC1L1,file="NPC1L1.csv")

PSCK9<-subset(exp_LDL,POS_b37<55630525)

PSCK9<-subset(PSCK9,POS_b37>55405221)

a=clump_data(PSCK9,clump_kb = 10000,

clump_r2 = 0.1)

PSCK9<-merge(a,PSCK9,by="SNP")

write.csv(PSCK9,file="PSCK9.csv")

CETP<-subset(exp_LDL,POS_b37<57117757)

CETP<-subset(CETP,POS_b37>56895762)

a=clump_data(CETP,clump_kb = 10000,

clump_r2 = 0.1)

CETP<-merge(a,CETP,by="SNP")

write.csv(CETP,file="CETP.csv")

#select SNPs for HDL-C proxied drug targets

HDL<-fread("without_UKB_HDL_INV_EUR_HRC_1KGP3_others_ALL.meta.singlevar.results.gz")

HDL$P<-2*pnorm(-abs(HDL$EFFECT_SIZE / HDL$SE))

exp_HDL<-subset(HDL,P<5e-08)

head(exp_HDL)

colnames(exp_HDL)[colnames(exp_HDL)=="rsID"] <- "SNP"

colnames(exp_HDL)[colnames(exp_HDL)=="P"] <- "pval.exposure"

CETP<-subset(exp_HDL,POS_b37<57117757)

CETP<-subset(CETP,POS_b37>56895762)

a=clump_data(CETP,clump_kb = 10000,

clump_r2 = 0.1)

CETP<-merge(a,CETP,by="SNP")

write.csv(CETP,file="CETP.csv")

#use phenoscanner to find confounders

library(phenoscanner)

snp_data <- read.csv(file = "total SNP.txt")

snp_split <- split(snp_data, ceiling(seq_len(nrow(snp_data))/100))

result_df <- data.frame()

for (snp_group in snp_split) {

phenors_list <- list()

for (snp in snp_group) {

phenors <- phenoscanner(snpquery = snp)

if (!is.null(phenors[["results"]])) {

phenors_list <- c(phenors_list, list(phenors[["results"]]))

}

}

tryCatch({

result_group <- do.call(rbind, phenors_list)

result_df <- rbind(result_df, result_group)

}, error = function(e) {cat("Error: Skipping group with inconsistent number of columns.\n")})

}

write.csv(result_df, file = "confounders.csv", row.names = FALSE)
